# Students' perceptions of online learning in higher education during COVID-19: an empirical study of MBA and DBA students in Egypt

**DOI:** 10.1186/s43093-022-00159-z

**Published:** 2022-10-01

**Authors:** Cherine Soliman, Doaa Salman, Gaydaa Osama GamalEldin

**Affiliations:** 1 Arab Academy for Science, Technology and Maritime Transport, Smart Village Campus – B 2401 – 6 October, Giza, Egypt; 2 Department Faculty of Management Sciences, October University for Modern Sciences and Arts, Cairo, Egypt

**Keywords:** Faculty satisfaction, e-learning, Online education, Faculty factors

## Abstract

Targeting to evaluate the analytical rigour of empirical research in management education, this study's goal is to find out how students felt about the sudden shift to online education. As well as to provide an assessment of online education performance in higher education from the students’ perception was it a success or a failure, or a path for change based on the findings? The study also considers the peculiarities of the Egyptian higher education system as well as the students’ environment, capabilities and limitations. An online questionnaire was used to survey 625 MBA and 41 DBA students. Results show that students’ satisfaction with online education is influenced by several factors, including their resources and talents. Student initiative was discovered to play a moderating role in the effects of student, instructor, and institution factors on students' satisfaction with online education. This research is being carried out during the COVID-19 outbreak to see how online instruction affects student achievement.

## Introduction

As the World Health Organization (WHO) announced that the world was officially experiencing a global pandemic in March 2020, governments followed the announcement with multiple measures, varying in severity and speed of response, from full lockdown to more simple versions of curfews.

The context forced businesses across all sectors to suddenly shift to online mode. The educational sector as a vital and critical sector was no exception. The entire educational system, at the school level and university level with both undergraduate and postgraduate, announced an emergency e-learning mode. The global context was unable to clearly state the timeline of the crisis. Based on the already existing infrastructure, with all its variation from one institution to another the educational sector executed the online mode with what was available to it and what was available to the students at home. As the crisis continued the educational institutions found themselves in the need to improve and invest in their technological infrastructure, software development, online educational policy and staff training.

While technology integration has always promised benefits these unique circumstances offer a great opportunity to study its true advantages and disadvantages. The objective of the paper is to further analyse the unique experience that the entire globe has lived in common. Capitalize on the experience in terms of success stories and failure experiences. The underlying of success or failure with the particularity and uniqueness of each environment. Hopefully leading to a roadmap on how to digitalize in favour of education with a focus on the student experience.

To answer the above interest, we start the paper with a literature review section on e-learning history. Then, we detail the fieldwork in terms of the organization of the study and environmental considerations. Next, we explain the methodology used in data collection and analysis, followed by the presentation of the results and analysis leading to the recommendations and limitations section to conclude the paper.

## Literature review

Since the launch of the first undergraduate and postgraduate online programs in 1989 – in Egypt, online education had its advocates and critics. Research in the educational field had been directed towards the exploration of online educational processes, to identify their strengths, weaknesses, and barriers, while offering solutions to challenges and facets of development when adopting online modes of study. In the following subsections, previous studies addressing the strengths and weaknesses of online learning are discussed, followed by a discussion of the abrupt transition from face-to-face learning to online learning amidst the COVID-19 pandemic. Then, a presentation is provided of the learning theories on which this study’s conceptual framework is built to assess the effectiveness of the COVID-19-prompted switch to online education in postgraduate programs offered in Egypt.

### Strengths of online education

There is strong evidence in the research [3, 7, 22] that online education gives students this kind of freedom. Researchers have also begun to investigate the flexibility that online education may provide for faculty in juggling their job and home lives [18, 19]. The viability of its business model is credited with its expansion, cost-effectiveness [34], flexibility, convenience, and accessibility—in terms of time and place [8, 20, 27]. This mode of study was also found attractive for the enhanced communication it offers between instructors and students and enhanced student interactions through discussion forums [36].

### Weaknesses of online education

Despite the global growth of online education, it had been extensively criticized in the literature and practice. While some online faculty members do feel more flexible, others believe that teaching online is "work-intensive and time-consuming, which can quickly lead to burnout." Other researchers have discovered that some online professors are concerned about flexibility, workload, and time needs [4, 31]. Moreover, it has been criticized for infrastructural barriers [28, 32] to students, instructors, as well as institutions [9] caused by the lack, inappropriateness, or poor management of the required hardware, software and connectivity [6].

Another aspect for which online education is criticized is the lack of proper training for both instructors and schools [28]. This lack of training contributes to the discomfort of instructors towards teaching online, computer anxiety, and fear of technology, especially among older generations [6]. There are also psychological and behavioural aspects by which online education is found less effective than face-to-face learning. When participating in online education, students feel isolated [24]. This isolation may lead to a lower sense of belonging [29] and impacts student discipline [26], sense of responsibility, time management, and motivation [23]. This is because working with others increases involvement by sharing ideas which stimulate critical thinking and deepened understanding [13].Online education is also criticized, by researchers and in practice, integrity issues not encountered in face-to-face learning [16].

### Switching to online education amid the COVID-19 pandemic

COVID-19 speeded the switch to online learning in an unprecedented way, without preparation or pre-testing [12]. The transition was rapid, with a limited time to plan or adapt. Courses originally planned to be delivered face-to-face were forced toward online delivery in a short time. Students, instructors, and institutions were required to adapt quickly. This raised concerns about the impact of this switch on the effectiveness of the educational process which might have been compromised to avoid total disruption of education. Online students' characteristics, attitudes, and outcomes have been the subject of a substantial amount of research [5, 14].

In an attempt to compare the effectiveness of online education to that of the originally adopted face-to-face mode, [29] performed a study based on a survey of economics and business administration instructors and students from universities in 13 European countries. Their sample consisted of 114 instructors and 248 students. Their results showed that students perceived higher effectiveness of online learning mainly attributed to the flexibility it offers to manage time, claim more responsibility, and receive continuous feedback. However, students perceived that the experience offer less interaction accompanied by an increased sense of isolation. The overall results showed that online learning is not perceived to maintain the quality of education offered by its face-to-face counterpart except for two aspects, namely, communication of high expectations to students and the time they spend preparing for a course. Decreased effectiveness was attributed to poorer instructor-student communication, poorer student–student collaboration, less active learning, deferred or lack of feedback, and less respect for differences among students. However, it is worth noting that the methods used in those universities were based on passive delivery and reduced interaction.

### Theories of learning effectiveness

To determine the dimensions which serve as proxies for the effectiveness of the learning process, particularly the online learning process stimulated by the COVID-19 pandemic, learning theories were reviewed to indicate the ones most appropriate to the online learning process.

In 1953, Skinner [35] developed the behaviourist learning theory which states that students learn while acting as passive participants when knowledge is transferred by an instructor. This learning approach was found appropriate for the transfer of objective knowledge [2]. Driven by the need to account for learning processes that are suitable for non-absolute knowledge transfer, the cognitive learning theory was then developed by Gagne in 1984 [15]. This theory states that learning takes place when learners are active participants, discovering, inquiring, and finding answers on their own.

As part of the development of learning processes, the contextual dimension started to be considered an essential component of effective learning processes. In 1987, Chickering and Gamson developed a taxonomy for the effectiveness of the educational process that consisted of 7 principles. These principles are (1) encouragement of instructor-student communication, (2) development of student–student cooperation, (3) use of active learning techniques, (4) offering timely feedback, (5) emphasis on student time on task, (6) communication of high expectations, and (7) respect for student differences. These principles are built on 50 years of research on teaching and learning and were found to lead to multiplied effects when applied together [13]. The study of Portela et al. [29] on the effectiveness of online education during COVID-19 was based on these seven principles.

In 1997, Boyle [11] developed the constructivist learning theory which takes learners’ social, cultural, and contextual conditions into consideration, by which they construct knowledge through experience. Constructivist learning is considered central to online learning due to its increased dependence on the learner’s conditions and efforts [37]. Within this framework, three theories were developed as extensions: collaborative learning, cognitive information processing, and facilitated learning. The collaborative learning theory emphasizes the role played by collaboration and sharing between learners and instructors. The cognitive information processing theory states that learning takes place through cognitive processes such as attention and encoding, storing and retrieving knowledge, which is promoted through course design [10]. Based on the facilitated learning model, learners consider new ideas when the learning environment encourages that [21]. This is established through institutional support.

The growing literature on the relevance of faculty attitudes for micro-level outcomes, or implications for specific people, like the learning and satisfaction of online students, and performance and retention of faculty teaching online, as well as macro-level outcomes, or implications for colleges and universities, like the success or failure of their e-learning initiatives, sparked our interest in studying faculty satisfaction in the context of online higher education [25, 38]. In 2021, Tsang et al. attempted to identify the predictors of online education effectiveness based on the constructivist learning theory and its extensions. Their analysis was based on a survey of 409 undergraduate students from 11 universities in Hong Kong. They hypothesized that perceived learning outcomes and student initiative could serve as proxies for learning effectiveness and would lead to student satisfaction. Their findings showed that student–student collaboration and course design were determinants of perceived learning outcomes and instructor-student communication was a determinant of student initiative. University support had no significant relationship with either perceived learning outcomes or student initiative.

The current study uses the model developed by Tsang et al. [37] to assess the effectiveness of online education resorted to during the COVID-19 pandemic. The study aims to contribute to the literature first by offering an application of the adopted model to measure the effectiveness of online learning effectiveness. Second, the current study not only offers an evaluation of the learning process but also determines the specific aspects that contribute to the effectiveness or ineffectiveness of the process. Third, the study also provides an evaluation of the online education process specifically within the graduate studies context. Focusing on this context is key because it is expected that the effectiveness of such a mode of education would significantly differ between undergraduates and postgraduates for two reasons, both based on the constructivist learning approach.

The social, cultural, and contextual conditions of postgraduates are different from those of undergraduates. Also, students enrolled in postgraduate business programs come from diverse backgrounds such as business, engineering, medicine, pharmacy, arts, and law. This paves the way for generalizable conclusions. Finally, to the researchers’ best knowledge, an assessment of the effectiveness of online education in postgraduate studies in Egypt has not taken place to date.

## Context of the case understudy

To respect the anonymity of the institution in which we have conducted our research all we can mention is that it has been serving the higher education sector in Egypt for 50 years today and offers more than 18 specializations from diplomas, to master to doctoral degrees, in both the professional and academic arena.

We focused our search on two degrees: the Master of Business Administration “MBA”, and the Doctorate of Business Administration “DBA”. The reason and objective behind this choice are the large numbers of students enrolled in these two programs which would present reliability to the findings and results.

As the lockdown was announced, this school was one week away from Fall 2019 end-of-semester exams, and amid heavy Spring 2020 recruitment. Strategic decisions were taken to resume with no interruption to the student schedule and the exams were carried out smoothly online. As the spring 2020 semester was announced to be conducted online, out of the 300 applicants 150 only decided to join online.

This 50–50% structure was rather a positive indicator for very logical reasons: the pessimistic economic outlooks on a global scale, a long-standing sceptical perception of the Egyptian educational institutions and society toward the online degree and questionable infrastructure, technological know-how among students and professors are also valid concerns and limitations.

**Fig. 1:**
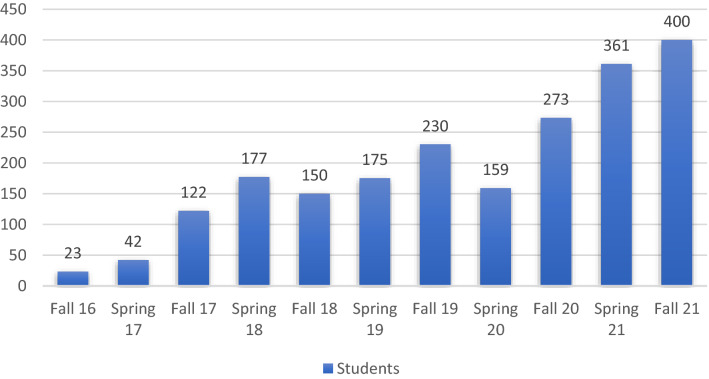
Student enrolment per semester

It is important to point out, that while only 150 candidates joined the online experience in Spring 2020, in the following semester we witnessed a remarkable increase in the enrollment numbers as represented in Fig. 1.

The school invested in ongoing surveys to enhance its understanding of what was going on which resulted in the development of the following questions:

1. Was online education a success or failure in terms of delivering educational content and experience in higher education?
2. Was online education a success or failure in enabling students’ social experience and professor rapport?
3. What is the impact of infrastructure and technological readiness on the online educational experience as an environmental factor?
4. What are the factors that had the greatest impact on the online educational experience?
5. What can we draw as lessons learnt from the student's perception of this experience and how to transform this into a roadmap for improvements?

## Research methodology

**Fig. 2:**
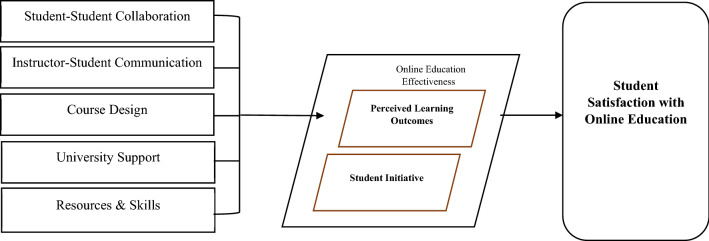
Online education conceptual framework

This study used a quantitative descriptive survey method to find out how students felt about their teachers' online instruction by giving them a questionnaire and using automated numerical computation to generate data. The focus of this study is student satisfaction with online education (outcome variable), mediated by perceived learning outcomes and student initiative. Based on the model evaluated by Tsang et al. [37], the predictor variables are student–student collaboration, instructor-student communication, course design, and university support. Based on the literature’s emphasis on the role played by technology-related aspects in the effectiveness of online education, a fifth predictor variable was added to the model, required resources and skills. Figure 2 illustrates the study’s conceptual framework. Based on this conceptual model, and to answer the research questions, the following hypotheses were developed:

H1: Student–student collaboration positively affects student satisfaction with online education.

H2: Instructor-student communication positively affects student satisfaction with online education.

H3: Course design positively affects student satisfaction with online education.

H4: University Support positively affects student satisfaction with online education.

H5: Availability of resources and skills positively affects student satisfaction with online education.

H6: Perceived learning outcomes activate a mediating mechanism in the effect on student satisfaction with online education.

H7: Student initiative activates a mediating mechanism in the effect on student satisfaction with online education.

### Method

For this study, an online survey was conducted, using a questionnaire with high reliability. Student–student collaboration, instructor-student communication, course design, and university support are the predictive variables, according to Tsang et al. [37].

However, some adjustments were made to certain questions, and a few were added particularly of our interest in the Egyptian context, for which we have repeated the questionnaire reliability test to ensure the robustness of the findings and results.

The survey was sent to students, targeting those that have experienced both classrooms as well as online education. We used SurveyMonkey to develop and dispatch the questionnaire. The population consisted of both males and females, with an age group from 22 to 55 years; all Great Cairo citizens, diversified working sectors and educational backgrounds.

The questionnaire is divided into four major areas of investigation and again divided into eight sections. The first focus was investigating the platforms and technological impact on the educational experience; the second focus was investigating the quality of the course content online; the third focus was investigating the student-professor experience; the final focus was investigating the impact of the environment particularly on Egypt on the overall experience. These are the different angles, below we explain section by section the questions' values and objectives.

Basic participant identification of program and gender only was required, the remaining participant identifications were kept anonymous hence it does not add or deduct the value of the survey.

The *first section* is dedicated to the assessment of university support. Five items were targeted: policy quality, visibility, communicability and impact from the students’ perception and experience.

*The second section* is rather environmental, with four items with a focus on students’ possession of adequate technology devices, stable internet connection and most importantly the digital knowledge to adapt and integrate online tools and platforms with sufficient confidence.

Section three was testing for student–student interactions: students’ influence, interaction, and network.

Section four assessed professor-student dialogue, with four items: its frequency, its impact, and how it translates to content and course understanding.

Next, section five focused on course design, with five items: course objective and material communication, module logical organization, intellectual stimulation, and then course assessment tools relevance.

Section six, consisting of four items, assessed the perceived learning outcome, with a focus on clear comparison, between online and face-to-face classroom experience in terms of understanding and assimilation of the course content and perceived quality.

The last section, consisting of two items only, assessed the level of satisfaction and education experience success from the online experience.

Overall, the questionnaire was designed to consume no more than three minutes to fill. A 5-point Likert scale increases the participant's probability to start and finish the questionnaire.

The number of completed surveys was 853. However, to include only those responses that were completed attentively, a speed factor was calculated for each respondent by dividing the time spent to complete the survey by the median time to complete the survey. Cases with speed factors higher than three were excluded from the sample, leading to 666 accepted responses.

## Results and discussion

To assess the reliability of the measurement tools, Cronbach’s alphas were calculated for each construct. As shown in Table 1, Cronbach’s alphas of the constructs showed acceptable internal consistency, with all alphas greater than 0.7.

**Table 1:** Constructs reliability

Constructs reliability		
Construct	No. of items	*⍺*
University support	5	0.924
Resources and skills	4	0.723
Student–student collaboration	4	0.948
Instructor-student Communication	4	0.972
Course design	5	0.945
Perceived learning outcomes	4	0.955
Student initiative	3	0.839
Student satisfaction	2	0.965

Descriptive statistics showed that the sample was composed of 41 DBA students, and 625 MBA students; 73.1% of them are males and 26.9% are females. Table 2 shows the mean and standard deviation of responses on the instructor, institution, and student determinants as well as perceived learning outcomes, student initiative, and student satisfaction.

**Table 2:** Descriptive statistics

Construct	Mean	SE
University support	4.27	0.86
Resources and skills	3.97	0.68
Student–student collaboration	3.88	1.16
Instructor-student communication	4.08	1.07
Course design	4.20	0.89
Perceived learning outcomes	3.82	1.24
Student initiative	3.62	1.13
Student satisfaction	4.32	1.07

To assess the effect of student–student collaboration, instructor-student communication, course design, and university support on student satisfaction with online education, correlation and multiple regression analyses were used. The correlation matrix in Table 3 shows that, at the 0.05 significance level, the program has a weak correlation with perceived learning outcomes and student initiative, with less student initiative and lower perceived learning outcomes among DBA students compared to MBA students. Gender showed no significant correlations with any of the variables. The highest significant correlations were between student–student collaboration and instructor-student communication and perceived learning outcome; instructor-student communication and each course design, perceived learning outcome, and overall satisfaction; course design and overall satisfaction; and perceived learning outcomes and overall satisfaction.

**Table 3:** Correlation Matrix

	Program	Gender	US	RS	SS	IS	CD	PLO	SI	OS
Program	1									
Gender	− 0.010	1								
US	− 0.052	0.048	1							
RS	− 0.010	− 0.023	.469^**^	1						
SS	− 0.047	0.028	.612^**^	.457^**^	1					
IS	− 0.063	0.063	.656^**^	.524^**^	.895^**^	1				
CD	− 0.032	0.025	.716^**^	.536^**^	.776^**^	.846^**^	1			
PLO	− 082^*^	0.038	.545^**^	.469^**^	.831^**^	.842^**^	.781^**^	1		
SI	− 087^*^	0.047	.378^**^	.284^**^	.571^**^	.572^**^	.555^**^	.580^**^	1	
OS	− 0.037	0.032	.640^**^	.551^**^	.790^**^	.841^**^	.822^**^	.820^**^	.512^**^	1

*Correlation is significant at the 0.05 level (2-tailed)

**Correlation is significant at the 0.01 level (2-tailed)

*US* University support, *RS* resources and skills, *SS *student–student collaboration, *IS* instructor-student communication, *CD* course design, *PLO* perceived learning outcome, *SI* student initiative, *OS* overall satisfaction

After testing the assumptions of multiple regression, the analysis was run to examine the effect of university support, resources and skills, student–student collaboration, instructor-student communication, and course design on student satisfaction with online education, controlling for program and gender. The model summary showed an R squared equal to 0.874, which implies that university support, resources and skills, student–student collaboration, instructor-student communication, and course design explain 87.4% of the variance in student satisfaction with online education. As shown in Table 4, the results of the analysis showed that resources and skills, student–student collaboration, instructor-student communication, and course design have a significant effect on student satisfaction with online education, while university support did not show significant effects at the 5% level.

**Table 4:** Coefficients: OS

Dependent variable: overall satisfaction					
	B	Std. error	Beta	*t*	Sig
(Constant)	− 0.152	0.152		− 1.000	0.318
Program	0.010	0.036	0.005	0.288	0.774
Gender	− 0.005	0.046	− 0.002	− 0.107	0.915
US	0.043	0.034	0.034	1.240	0.216
RS	0.167	0.036	0.107	4.641	0.000
SS	0.146	0.040	0.158	3.692	0.000
IS	0.347	0.051	0.348	6.774	0.000
CD	0.389	0.047	0.323	8.208	0.000

*US* University support, *RS* resources and skills, *SS *student–student collaboration, *IS* instructor-student communication, *CD* course design

Based on this analysis, hypotheses H1, H2, H3, and H5 are accepted, while H4 is rejected. To test H5 and H6 to determine whether perceived learning outcomes and student initiative stimulate mediating mechanisms, path analysis was performed. The starting point was to examine the effect of the five predictor variables on the mediating variables of perceived learning outcome and student initiative, controlling for program and gender.

The results (Table 5) showed that university support, student–student collaboration, instructor-student communication, and course design had significant relations with perceived learning outcomes, while resources and skills did not show significant results. The model summary showed an R squared equal to 0.76, implying that the predictor variables explained 76% of the variation in perceived learning outcomes. However, the negative university support coefficient shows that increased university support for online education did not lead to better-perceived learning outcomes as compared to face-to-face education.

**Table 5:** Coefficients: PLO and SI

	B	Std. Error	Beta	*t*	Sig
*Dependent variable: perceived learning outcome*					
(Constant)	− 0.361	0.179		− 2.021	0.044
Program	− 0.087	0.042	− 0.040	− 2.090	0.037
Gender	0.016	0.054	0.006	0.299	0.765
US	− 0.166	0.040	− 0.114	− 4.095	0.000
RS	0.057	0.042	0.031	1.341	0.180
SS	0.409	0.047	0.379	8.784	0.000
IS	0.380	0.060	0.327	6.324	0.000
CD	0.383	0.056	0.273	6.898	0.000
*Dependent variable: student initiative*					
(Constant)	1.152	0.262		4.392	0.000
Program	− 0.118	0.061	− 0.060	− 1.924	0.055
Gender	0.065	0.079	0.026	0.819	0.413
US	− 0.112	0.059	− 0.085	− 1.888	0.059
RS	− 0.061	0.062	− 0.037	− 0.988	0.324
SS	0.277	0.068	0.284	4.057	0.000
IS	0.141	0.088	0.134	1.594	0.111
CD	0.381	0.082	0.300	4.672	0.000

*US* University support, *RS* resources and skills, *SS *student–student collaboration, *IS* instructor-student communication, *CD* course design

Online education

The results also showed that only student–student collaboration and course design had significant relations with student initiatives, while university support, resources and skills, and instructor-student communication did not show significant results. The model summary showed an R squared equal to 0.369, implying that the predictor variables explained 36.9% of the variation in student initiative.

The final step to run the path analysis was to examine the effect of each perceived learning outcome and student initiative on student satisfaction with online education. The results (Table 6) showed that both perceived learning outcomes and student initiative have a significant effect on student satisfaction with online education. The model summary showed an R squared equal to 0.675, implying that the predictor variables explained 67.5% of the variation in student satisfaction with To perform path analysis, the PROCESS Macro of SPSS was used. First, the effect of university support on overall satisfaction through perceived learning outcomes and student initiative was assessed. The results showed no direct effect of university support on student satisfaction (LLCI = 0.0201, ULCI = 0.1503), but an indirect effect exists through perceived learning outcome only (BootLLCI = − 0.0797, BootULCI = − 0.0191).

**Table 6:** Coefficients: effect of PLO and SI on OS

Dependent variable: overall satisfaction					
	B	Std. Error	Beta	t	Sig
(Constant)	1.545	0.087		17.759	0.000
PLO	0.677	0.023	0.788	29.011	0.000
SI	0.052	0.026	0.055	2.017	0.044

*PLO* Perceived learning outcome, *SI* student initiative

Second, the effect of resources and skills on overall satisfaction through perceived learning outcomes and student initiative was assessed. The results showed a direct effect of resources and skills on student satisfaction (LLCI = 0.0827, ULCI = 0.2172), with no indirect effect through perceived learning outcome and student initiative (BootLLCI = − 0.0797, BootULCI = − 0.0191).

Third, the effect of student–student collaboration on overall satisfaction through perceived learning outcomes and student initiative was assessed. The results showed no direct effect of student–student collaboration on student satisfaction (LLCI = − 0.0371, ULCI = 0.1202), but an indirect effect exists through perceived learning outcomes only (BootLLCI = 0.0700, BootULCI = 0.1634).

Fourth, the effect of instructor-student communication on overall satisfaction through perceived learning outcome and student initiative was assessed. The results showed a direct effect of instructor-student communication on student satisfaction (LLCI = 0.1481, ULCI = 0.3449) and an indirect effect through perceived learning outcome only (BootLLCI = 0.0595, BootULCI = 0.1553).

Finally, the effect of course design on overall satisfaction through perceived learning outcomes and student initiative was assessed. The results showed a direct effect of course design on student satisfaction (LLCI = 0.2012, ULCI = 0.3859) and an indirect effect through perceived learning outcome only (BootLLCI = 0.0561, BootULCI = 0.1637).

The results indicate that perceived learning outcomes play a mediating role in the effect of student, instructor, and university determinants on student satisfaction with online education. Hence, H6 is accepted. However, student initiative does not activate a mediating mechanism in the effects on student satisfaction with online education. Hence, H7 is rejected. It is indicated that the student initiative’s role may rather be a moderating one. To test this, regression analysis was run including six interaction terms between student initiative and each university support, resources and skills, student–student collaboration, instructor-student communication, course design, and perceived learning outcomes. The new model summary, including the six interaction terms, perceived learning outcome, and student initiative, showed an R Squared equal to 0.898, implying an improvement of variance explained to 89.8% when interaction terms were included. As shown in Table 7, interaction terms between student initiative and instructor-student communication, course design, and perceived learning outcome showed significant effects at the 5% level, implying the moderating effect of student initiative.

**Table 7:** Coefficients: OS with Interactions

Dependent variable: overall satisfaction with interactions					
	B	Std. Error	Beta	*t*	Sig
(Constant)	− 1.766	0.311		− 5.670	0.000
Program	0.041	0.033	0.022	1.250	0.212
Gender	0.006	0.042	0.003	0.144	0.885
US	0.202	0.094	0.162	2.150	0.032
RS	0.264	0.100	0.169	2.629	0.009
SS	0.076	0.126	0.082	0.602	0.547
IS	− 0.226	0.168	− 0.226	− 1.345	0.179
CD	0.571	0.152	0.474	3.746	0.000
PLO	0.625	0.113	0.728	5.521	0.000
SI	0.609	0.098	0.642	6.211	0.000
Int1	− 0.032	0.025	− 0.191	− 1.251	0.211
Int2	− 0.039	0.028	− 0.202	− 1.391	0.165
Int3	− 0.005	0.036	− 0.037	− 0.152	0.879
Int4	0.130	0.050	0.870	2.607	0.009
Int5	− 0.106	0.046	− 0.666	− 2.302	0.022
Int6	− 0.098	0.033	− 0.698	− 2.978	0.003

US = University support, RS = resources and Skills, SS = student–student collaboration, IS = instructor-student communication, CD = course design, PLO = perceived learning outcome, SI = student initiative, Int1 = SI*US, Int2 = SI*RS, Int3 = SI*SS, Int4 = SI*IS, Int5 = SI*CD, Int6 = SI*PLO

**Fig. 3:**
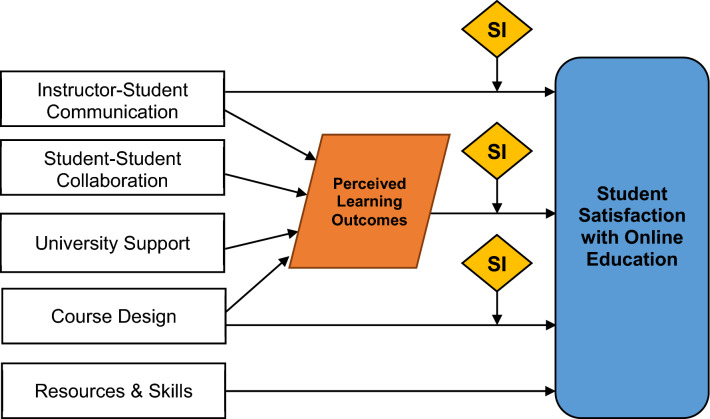
Modified conceptual model

Based on this analysis, the relations between the predictor variables, mediating variables, moderating variables, and outcome variables can be depicted as shown in Fig. 3.

Although the online style of classes was their first experience during the COVID-19 outbreak, the students agreed that online instruction was beneficial to them [1, 17, 30, 33]. On the other hand, demographic characteristics play an important influence in determining how well an online course performs.

## Implication and recommendations

The above revealed that the participants found online education with advantages and limitations.

Though it was their first time to experience online learning however students has found it to be valuable since it ensured their program continuity despite the pandemic. Students though it to be manageable and convenient in terms of time management, less driving time, more accommodating to their other professional and personal (family) obligations. The online mood also made their student administrative task rather easy. The experience encouraged the student to engage more in self-directed learning and creating different moods of mutual students support amongst them.

Students has identified that during online learning they were not able to acquire the practical side of the courses yet there was rather a limitation of focus on the knowledge factor; a clear lack in the ability from professors side to assess students’ understanding during online lecture; problem with span of attention due to the long hours of online activities (both professional and educational);

Based on the above results we were able to develop the following recommendation to the higher education sector working in Egypt with the objective to help enhance the online education experience:

1. More research and investigation is needed to further comprehend how to optimize on the mode and modalities of online learning, more factors needs to be investigated in terms of context particularity.
2. Universities need to invest in the development of education policy, course design, curricula that are more technology based friendly which in its turn would make online education more effective.
3. The participants’ results indicated that the hybrid (blended) learning option could be a better context for the post graduate students in Egypt, creating a balance between online education and face to face mode of learning.
4. Continuous technology education is needed for both the faculty and the students to enhance their chances in online education optimization
5. Universities need to invest in high quality software and infrastructure.
6. All the above will only be possible if and when University strategy and long term objective embrace technology foundation amongst its priorities

We support that these recommendations can support the higher education sector in Egypt.

## Conclusion and contribution

Concerning the assessment of whether online education was a success or failure based on student satisfaction, the results reflect high student satisfaction reflected in a mean above 4.0. Favourable perceptions toward university support, instructor-student communication, and course design were also found. Less favourable perceptions were found toward student–student collaboration, perceived learning outcomes, and student initiative. This is consistent with previous studies highlighting behavioural aspects that hinder the outcomes of online education, such as a sense of isolation [24], lower sense of belonging [29], sense of responsibility, time management, and motivation [23].

Resources and skills also showed less favourable perceptions, consistent with the view that infrastructural barriers introduce themselves in online education experiences [6, 28, 32].

The factors that were found to have the greatest impact on the online educational experience are instructor-student communication and course design. Both university support and student–student collaboration were found to only have an indirect impact on student satisfaction through perceived learning outcomes. Resources and skills were found to directly affect student satisfaction. Instructor-student communication and course design were both found to have both direct and indirect effects on student satisfaction with online education. This means that online constructive interactions with instructors and courses with clear objectives, structure, interesting material, challenges, and assessment tools enhanced the student experience and perceived learning outcomes, which again added to student satisfaction.

Finally, this study provides two enhancements to the model developed and evaluated by Tsang et al. [37]. First, a fifth predictor variable was added to the model, resources and skills, which were shown to have a direct positive impact on students’ satisfaction with online education. Second, student initiative, identified as a mediating variable by Tsang et al. [37], was found to rather have a moderating role in how student, instructor, and institution determinants affect students’ satisfaction with online education. In this sense, students who possess the characteristics to take initiative to make the best use of the online experience are more satisfied.

Overall, online education did encourage the educational institution to further their student-centered experience. However launched in an emergency mood all parties involved in the activity have rather found it to be manageable. The current findings are encouraging to recommend to school to further invest in online learning to overcome obstacles and limitations. There is a need to continuously develop the faculty on online learning modalities, further adapt courses to online methodology to ensure results; invest in high quality software.

## Limitations and future studies

While the study proves reliability through the number of candidates participating in the survey, the rigorous measures of eliminations in the sample, the validity value of the questionnaire, and the literature recommendation of the model used here yet it is important to point out that: further elements in the e-learning can and need to be studied such as cultural implications, generations differences; government support reality from policies to infrastructure, management philosophy readiness in developing countries among other factors.

## Data Availability

The datasets used and/or analysed during the current study are available from the corresponding author on reasonable request.

## Abbreviations

DBA: Doctorate of business administration
MBA: Master of business administration
WHO: World Health Organization
