# Correction: Students’ perceptions of online learning in higher education during COVID-19: an empirical study of MBA and DBA students in Egypt

**DOI:** 10.1186/s43093-022-00178-w

**Published:** 2022-12-19

**Authors:** Cherine Soliman, Doaa Salman, Gaydaa Osama GamalEldin

**Affiliations:** 1 Arab Academy for Science, Technology and Maritime Transport, Smart Village Campus – B 2401 – 6 October, Giza, Egypt; 2 Department Faculty of Management Sciences, October University for Modern Sciences and Arts, Cairo, Egypt

**Correction to: Future Business Journal (2022) 8:45** **https://doi.org/10.1186/s43093-022-00159-z**

Following publication of the original article [1], the authors identified an error in the author name of Gaydaa Osama GamalEldin.

The incorrect author name is: Gaydaa Osama Gamal el din

The correct author name is: Gaydaa Osama GamalEldin

The author group has been updated above and the original article [1] has been corrected.
